# Psychological and Emotional Impact of COVID-19 Pandemic on People Living with Chronic Disease: HIV and Cancer

**DOI:** 10.1007/s10461-022-03638-0

**Published:** 2022-03-06

**Authors:** Emanuele Focà, Chiara Fornari, Stefania Arsuffi, Maria Chiara Vetrano, Stefano Calza, Stefano Renzetti, Silvia Copeta, Alfredo Berruti, Francesco Castelli, Silvia Compostella, Eugenia Quiros-Roldan

**Affiliations:** 1 Unit of Infectious and Tropical Diseases, Department of Clinical and Experimental Sciences, University of Brescia and ASST Spedali Civili Hospital, Brescia, Italy; 2 Unit of Biostatistics and Bioinformatics, Department of Molecular and Translational Medicine, University of Brescia, Brescia, Italy; 3 Department of Medical and Surgical Specialties, Radiological Sciences, and Public Health, Medical Oncology, University of Brescia at ASST Spedali Civili, Brescia, Italy; 4 Unit of Infectious Diseases of ASST Spedali Civili Hospital, Brescia, Italy

**Keywords:** COVID-19, Psychological effects, Resilience, Chronic diseases

## Abstract

People living with chronic disease (PLWCD) are the frailest category, both for the risk of severe COVID-19 illness and for the impact on the care continuum. Aim of this study was to analyze coping strategies and resilience in people living with HIV (PLWH) compared to people living with oncological diseases (PLWOD) during COVID-19 pandemic. We administrated an anonymous questionnaire, which explored the emotional experience, the demographic factors linked to a COVID-19-related stress syndrome, the patient’s perception about the adequacy of clinical undertaking from the hospital and the resilience. We analyzed 324 questionnaires. There were no significant differences in prevalence of psychological distress among the whole cohort; however, PLWOD were calmer, less troubled, and more serene than PLWH. Moreover, PLWH smoked more, ate more, and gained more weight than PLWOD. Most patients didn’t feel lonely and continued to take pleasure from their activities. No differences in resilience were found between the groups. In the whole cohort lower levels of resilience were found in patients that were unemployed, with history of psychological disorders and in those who experienced more feelings of anger, anxiety and concern. In our study, patients seemed to preserve their well-being, and to activate adaptive coping during the pandemic.

**Supplementary Information:**

The online version contains supplementary material available at 10.1007/s10461-022-03638-0.

## Introduction

SARS-Cov-2 was discovered in Wuhan, China, in December 2019 and in short time caused an international health emergency [1–3]. The World Health Organization (WHO) declared the outbreak of a pandemic on March 11, 2020 [1]. COVID-19 caused the health systems distress around the world [4].

Italy and in particular Lombardy region suffered from an increasing number of cases with a high rate of intra-hospital mortality [5]. In the first phase in March–April 2020 the Brescia Hospital, a large tertiary hospital with 15,709 beds, was one of the referral Hub-Hospital according to the high number of hospitalized patients.

The COVID-19 pandemic changed the certainty, activated fear, and inducted feelings like loneliness and isolation [3, 6]. The containment measures limited the individual freedom [6] and the burden on mental health conditions increased [7–10]. People living with chronic disease (PLWCD) are the most frail social category, not only for the risk for severe COVID-19 illness but also for the impact of the changes in the self-management [6, 11, 12].

Furthermore, COVID-19 pandemic had the potential to destroy the continuum of care [13–16] and people’s lives [6, 17]. People living with HIV (PLWH) and people living with oncological disease (PLWOD) represent a vulnerable population that may potentially be at higher risk to have severe COVID-19 compared to general population [2, 16, 18, 19]. This risk in PLWCD is predicted on potential interactions between COVID-19, presence of immunodeficiency and the co-existence of comorbidities [2, 4, 18]. Moreover, the stress due to the above-mentioned factors and the containments measures, like lockdown, have had an impact on mental health conditions [8] both in general population and in chronic patients [6].

The resilience-related factors, such as the perceived social support, are protective factors for the management of the chronicity [20] and for mitigating health challenges and stressful situations [4]. Several studies conducted in PLWH showed that depression is associated with treatment failure [21, 22], lower CD4 + t-cell count [23, 24]; while anxiety is linked with disengagement from care [25, 26] and non-adherence to antiretroviral therapy [27, 28]. On the other hand, the resilience and coping strategies developed by PLWH may potentially be a protective factor compared to general population [16, 29]. Indeed, considering the past, they could have a deep knowledge of pandemics and their effects and know how managing life in this specific context and how to cohabit with the stigma [16, 30]. Moreover, such similarities between the COVID-19 and HIV pandemics are present: the absence of vaccination at the time of the study and the perception of isolation and fear [16, 29].

According to the syndemic framework, we considered the biopsychosocial perspective of health and disease to understand the effects of the pandemic in PLWCD [19]. A syndemic is defined as two or more epidemic events that interact synergically to produce an increased burden in a specific population [19, 31]. This perspective was mostly applied in PLWH [32]: the personal experience of COVID-19 pandemic is assumed to overlap with others health challenges due to HIV, including the disease itself, mental burden, and other infections [33].

Given that higher rates of mental health problems have been reported in general population in the setting of the pandemic [4], collecting psycho-social health data among PLWCD is important. PLWH may have higher score of resilience and that COVID-19 may generate a weaker psychological impact in the face of reduced social support [34, 35] and disadvantaged socio-economic conditions [19] than PLWOD.

The aim of the study is to describe the emotional experiences and the psychological characteristics (including resilience) of PLWCD, such as PLWH compared to PLWOD, during COVID-19 pandemic in order to gain clues for the medical staff and to make more effective the psychological, clinical and therapeutic interventions.

## Methods

This observational study was conducted from December 16, 2020 to February 19, 2021 at the HIV and oncological outpatient clinics of ASST Spedali Civili Hospital, Brescia, Italy. The study involved the administration of an *ad hoc* descriptive anonymous questionnaire and a resilience scale (RS) [36]. The questionnaire was dealt with as a structured interview to increase the quality of data and the accessibility. The compilation requires about 20 min. The questionnaire and the RS were proposed to the patients during one of the routine medical contacts at the Spedali Civili Hospital.

This study was approved by Ethical Board of Brescia Province (procedure: NP 4364). It was conducted in accordance with the guidelines and standards of the Declaration of Helsinki (2013) and with the principles of Good Clinical Practice. The participation was voluntary, and no written informed consent was needed. All the participants gave a verbal informed consent, participants that denied the consent were excluded.

### The Questionnaire

We have constructed a questionnaire (Supplemental Material) aimed to explore: the emotional experience and the stress due to the sanitary emergency of COVID-19 in PLWH and PLWOD; the patient’s perception about the adequacy of the clinical and psychological undertaking from the hospital during the COVID-19 pandemic; the clinical and demographic factors linked with a higher level of COVID-19 related stress (i.e. age, gender, disease).

The questionnaire was subdivided in clusters of questions: socio-demographic information and history, patients’ family network and perceived support, feelings and experiences during the quarantine, patients’ experience in their respective hospital clinic, management of the disease during the lockdown, patient’s opinions about Coronavirus and sources of information used. The compilation of the questionnaire requires about 15 min, and it includes both dichotomous questions which only have two possible responses, such as “Yes” or “No”, closed-ended questions and 3-points Likert-type scale (1 = not at all; 2 = a little; 3 = a lot).

### The Resilience Scale

Resilience has been defined as the personal dynamic characteristic that moderates the negative effects of stress, that promotes the positive adaptation and effective coping strategies [37, 38]. The Italian version of the RS [39] (Supplemental Material) was administered to identify the capability and effective resources of patients in order to manage the critical psychological issue during the pandemic. The compilation of the RS requires about 5 min.

### Statistical Analysis

Continuous variables were reported as mean with standard deviation (mean ± SD) and were compared across the groups using the nonparametric Kruskal–Wallis test. Categorical variables were summarized through frequencies and percentages and were compared across the groups using the Chi-squared test. The Exploratory Factor Analysis (EFA) was computed to investigate the latent variables underlying the questions of the questionnaire regarding the emotional experiences and the life quality during the lockdown. The factor loading threshold cut-off value was set at |0.32|. The factors that emerged from the EFA and demographic data were included in a linear regression model with the score obtained from the RS. Statistical significance level was set at α = 0.05; all statistical tests were two-tailed and 95% CI were computed for linear regression parameters. Statistical analysis was performed with R (version 4.0.2).

## Results

### Participant Characteristics and Socio-Economic Aspects

A total of 600 adults (≥ 18 years old. 315 PLWH vs. 285 PLWOD) were recruited for the study, after being admitted to the Hospital for the routine visits. 211 patients (35.2% of the total) refused to take part to the study: 143 patients with HIV (32.2% women) and 68 patients with oncological disease (42.7% women). Lack of time and will were the main causes of refusal. Furthermore, 65 (10.8%) questionnaires were excluded as were incomplete. Overall, 324 (54% of the total) were eligible for analyses: 167 PLWH and 157 PLWOD. PLWH were significant younger compared to PLWOD (H = 61.0, *p* < 0.001) and PLWH had a significant longer history of disease compared to PLWOD (χ^2^ = 135.7, *p* < 0.001) (Table 1).

**Table 1:** Participant characteristics

Recruitment: n (%)	PLWH (N = 315)	PLWOD (N = 285)	Total (N = 600)
Participants	167 (51.5%)	157 (48.5%)	324 (54%)
Refusal	143 (67.8%)	68 (32.2%)	211 (35.2%)
Interrupted	5 (7.7%)	60 (92.3%)	65 (10.8%)

Characteristics	Observed		Total (N = 324)	Kruskall-Wallis H Test	χ^2^ Test	*p* value
	PLWH (N = 167)	PLWOD (N = 157)				
Age				61.0		< 0.001**
Year: mean (sd)	51.0 (11.2)	61.6 (12.5)	56.1 (13.0)			
Sex: n (%)					17.2	< 0.001**
Male	114 (68.3%)	73 (46.5%)	187 (57.7%)			
Female	52 (31.1%)	84 (53.5%)	136 (42.0%)			
Others	1 (0.6%)	0 (0%)	1 (0.3%)			
Education:						
Year: mean (sd)	10.6 (3.9)	10.7 (4)	10.6 (3.9%)	0.02		0.880
Level of education: n (%)					7.9	0.048*
Primary school diploma	17 (10.2%)	26 (16.6%)	43 (13.3%)			
Middle school diploma	74 (44.3%)	49 (31.2%)	123 (38.0%)			
High school diploma	55 (32.9%)	65 (41.4%)	120 (37.0%)			
Degree	21 (12.6%)	17 (10.8%)	38 (11.7%)			
Nationality: n (%)					3.0	0.084
Italian	156 (93.4%)	153 (97.5%)	309 (95.4%)			
Foreigner	11 (6.6%)	4 (2.5%)	15 (4.6%)			
Diagnosis: n (%)					135.7	< 0.001**
< 5 years	17 (10.2%)	116 (73.9%)	133 (41.0%)			
≥ 5 years	150 (89.8%)	41 (26.1%)	191 (59.0%)			
Cohabitation during the interview: n (%)					12.5	< 0.001**
Single	48 (28.7%)	20 (12.7%)	68 (21.0%)			
With someone	119 (71.3%)	137 (87.3%)	256 (79.0%)			
Cohabitation during the quarantine (first wave): n (%)					12.0	< 0.001**
Single	42 (25.2%)	17 (10.8%)	59 (18.2%)			
With someone	125 (74.9%)	140 (89.2%)	265 (81.8%)			

Kruskall–Wallis H Test; Chi-Square χ^2^ Test

*p < 0.05; **p < 0.001

Additionally, from the socio-economic point of view, 153 patients (47.2%) were employed while 103 patients (31.8%) were retired. A greater number of PLWH than PLWOD were employed, while a significant greater number of PLWOD were retired (χ^2^ = 61.6, *p* < 0.001). At last, PLWH were significant more worried than PLWOD about economics and financial problems due to the pandemic (32.9% PLWH vs. 9.56% PLWOD; χ^2^ = 30.9, *p* < 0.001) (Table 2).

**Table 2:** Socio-economic aspects

Characteristics	Observed		Total (N = 324)	χ^2^ Test	*p* value
	PLWH (N = 167)	PLWOD (N = 157)			
Employment: n (%)				61.6	< 0.001**
Employed	103 (61.7%)	50 (31.8%)	153 (47.2%)		
Pensioners	24 (14.4%)	79 (50.3%)	103 (31.8%)		
Housewife	11 (6.6%)	17 (10.8%)	28 (8.6%)		
Unemployed	21 (12.6%)	4 (2.6%)	25 (7.7%)		
Other	8 (4.8%)	6 (3.8%)	14 (4.3%)		
Student	0 (0.0%)	1 (0.6%)	1 (0.3%)		
Habitation: n (%)				24.7	< 0.001**
Spacious house with garden	123 (73.7%)	146 (93.0%)	269 (83.0%)		
Three-room apartment	20 (12.0%)	9 (5.7%)	29 (9.0%)		
Two-room apartment	17 (10.2%)	2 (1.3%)	19 (5.9%)		
Studio apartment	7 (4.2%)	0 (0.0%)	7 (2.2%)		
Concern for economics problems: n (%)				30.9	< 0.001**
Not at all	83 (49.7%)	121 (77.1%)	204 (63.0%)		
A little	29 (17.4%)	21 (13.4%)	50 (15.4%)		
A lot	55 (32.9%)	15 (9.6%)	70 (21.6%)		

Chi-Square χ^2^ Test

*p < 0.05; **p < 0.001

Almost 25% of total patients referred to have a history of psychological problems without significant differences between groups. Depression was the most widespread psychological disorder among the groups. Furthermore, the majority of the patients (97.2%) reported to respect the care continuum during the pandemic. (Table S1 in supplementary materials).

### Information About COVID-19

Patients’ general knowledge about important aspects of COVID-19 pandemic was analyzed (Table 3). The majority of patients (87%) reported to be alert to the COVID-19-related symptoms, such as anosmia, ageusia, fever, cough, fatigue, and dyspnoea (86.2% of PLWH vs. 87.9% of PLWOD). Participants were aware about the recommended prevention methods. In particular, 150 patients (46.9%) sustained that facial masks are enough to protect from the virus while 139 patients (43.4%) reported that not only facial masks but also physical distancing and hand washing are necessary to protect from the infection.

**Table 3:** General knowledge

Characteristics	Observed		Total (N = 324)	χ^2^ Test	*p* value
	PLWH (N = 167)	PLWOD (N = 157)			
Pay attention to Covid-19 symptoms: n (%)				5.9	0.052
Not at all	5 (3.0%)	11 (7.0%)	16 (4.9%)		
A little	18 (10.8%)	8 (5.1%)	26 (8.1%)		
A lot	144 (86.2%)	138 (87.9%)	282 (87.0%)		
The facial masks are enough to protect yourself: n (%)				1.7	0.421
N-Miss	2	2	4		
Not at all	19 (11.5%)	12 (7.7%)	31 (9.7%)		
A little	73 (44.2%)	66 (42.6%)	139 (43.4%)		
A lot	73 (44.2%)	77 (49.7%)	150 (46.9%)		
Concern of being at risk: n (%)				8.1	0.018**
N-Miss	1	0	1		
Not at all	88 (53.0%)	66 (42.0%)	154 (47.7%)		
A little	35 (21.1%)	27 (17.2%)	62 (19.2%)		
A lot	43 (25.9%)	64 (40.8%)	107 (33.1%)		
Worsening of the chronic disease: n (%)				9.9	0.007**
N-Miss	1	2	3		
Not at all	83 (50.0%)	53 (34.2%)	136 (42.4%)		
A little	24 (14.5%)	21 (13.5%)	45 (14.0%)		
A lot	59 (35.5%)	81 (52.3%)	140 (43.6%)		
Vaccination: n (%)				2.7	0.253
As soon as possible	98 (58.7%)	105 (66.9%)	203 (62.7%)		
Wait to see the effects	48 (28.7%)	39 (24.8%)	87 (26.9%)		
Never	21 (12.6%)	13 (8.28%)	34 (10.5%)		

Chi-Square χ^2^ Test

*p < 0.05; **p < 0.001

Furthermore, the association between living with chronic disease and living during the pandemic was analyzed. When asked about living with chronic diseases while facing the risk of contracting COVID-19, most of the participants (47.7%) reported that they no longer consider themselves at higher risk than other people (53% of PLWH vs. 42% of PLWOD). However, a significant greater number of PLWOD than PLWH thought to be at risk (25.9% of PLWH vs. 40.8% of PLWOD; χ^2^ = 8.1, *p* = 0.018) and were worried about their clinical outcome (35.5% of PLWH and 52.3% of PLWOD; χ^2^ = 9.9, *p* = 0.007). Lastly, most of the participants (62.7%) would like to get the vaccination as soon as it was available (58.7% of PLWH vs. 66.9% of PLWOD).

In sum, most of the patients were careful about the symptoms of COVID-19 and they understood the importance of protecting themselves and reducing the diffusion of the virus: PLWOD were in general more aware and worried about the outcome of the COVID-19 infection than PLWH.

The 97.2% of patients were aware of the progress of the pandemic (Table S2 in supplementary materials). Patients consulted the COVID-19-related information from different sources, the most used ones were television, internet, and social media. Most of the patients (71.9%) reported that information obtained by the doctors were useful, while information provided by politicians were confused and catastrophic. 161 patients (49.7%) checked the news every 3–5 h and PLWOD consulted the COVID-19-related information significant more frequently than PLWH (42.5% of PLWH vs. 57.3% of PLWOD; χ^2^ = 7.5, *p* = 0.013).

### Social Support and Coping Strategies

Social and family networks were analyzed to identify the support received (Table S3 in supplementary materials). The 81.2% of participants reported to be very helped by the family in the everyday life both for the practical, psychological, and emotional aspects. They felt also protected to express their COVID-19-related concerns and thoughts to the family. Moreover, the majority of the participants (62%) reported not having feelings of solitude than usual during the quarantine. Indeed, during the pandemic, participants have managed to maintain good relationships with others, such as family and friends. Furthermore, the majority of the participants (73.6%) did not receive and search support from other patients with the same disease.

Three aspects of coping strategies were analyzed: the faith, the using of meditation techniques and the ability to take pleasure from their activities and hobbies. Only the 29.5% of patients received support by the faith (19.3% of PLWH vs. 40.5% of PLWOD; χ^2^ = 19.0, *p* < 0.001). Most of the participants (80.5%) did not use meditation techniques while the 71.3% continued to take pleasure from their activities and hobbies. Patients reported that found new activities, such as cooking, when their previous hobbies were suspended.

### Stress and Experiences

Emotional experiences of the participants were analyzed to understand the stress of the patients during the quarantine (Table 4). A greater number of PLWOD were significant calmer than PLWH (χ^2^ = 4.2, *p* = 0.039) and, on the other hand, a greater number of PLWH were more restless than PLWOD (χ^2^ = 6.0, *p* = 0.014). Overall, 254 patients (78.4%) referred to have lived peacefully (72.5% of PLWH vs. 84.7% PLWOD; χ^2^ = 7.3, *p* = 0.026).

**Table 4:** Stress and experiences

Characteristics	Observed		Total (N = 324)	χ^2^ Test	*p* value
	PLWH (N = 167)	PLWOD (N = 157)			
Feelings during the quarantine: n (%)					
Calm				4.2	0.039*
Yes	82 (49.1%)	95 (60.5%)	147 (45.4%)		
No	85 (50.9%)	62 (39.5%)	177 (54.6%)		
Indifferent				0.4	0.530
Yes	5 (3.0%)	3 (1.9%)	8 (2.5%)		
No	162 (97.0%)	154 (98.1%)	316 (97.5%)		
Sad				1.6	0.207
Yes	21 (12.6%)	13 (8.3%)	34 (10.5%)		
No	146 (87.4%)	144 (91.7%)	290 (89.5%)		
Sense of guilty				–	0.579
Yes	0 (0.0%)	0 (0.0%)	0 (0.0%)		
No	167 (100%)	157 (100%)	324 (100%)		
Powerless				2.5	0.116
Yes	23 (13.8%)	13 (8.28%)	36 (11.1%)		
No	144 (86.2%)	144 (91.7%)	288 (88.9%)		
Angry				0.02	0.887
Yes	10 (6.0%)	10 (6.4%)	20 (6.2%)		
No	157 (94.0%)	147 (93.6%)	304 (93.8%)		
Nerves				0.6	0.435
Yes	12 (7.2%)	8 (5.1%)	20 (6.2%)		
No	155 (92.8%)	149 (94.9%)	304 (93.8%)		
Troubled				6.0	0.014*
Yes	20 (12.0%)	7 (4.46%)	27 (8.33%)		
No	147 (88.0%)	150 (95.5%)	297 (91.7%)		
Worried				1.7	0.196
Yes	80 (47.9%)	64 (40.8%)	144 (44.4%)		
No	87 (52.1%)	93 (59.3%)	180 (55.6%)		
Anxious				1.6	0.209
Yes	26 (15.6%)	17 (10.8%)	43 (13.3%)		
No	141 (84.4%)	140 (89.2%)	281 (86.7%)		
Frightened				0.2	0.659
Yes	38 (22.8%)	39 (24.8%)	77 (23.8%)		
No	129 (77.3%)	118 (75.2%)	247 (76.2%)		
Panic				0.6	0.421
Yes	3 (1.8%)	5 (3.19%)	8 (2.5%)		
No	164 (98.2%)	152 (96.8%)	316 (97.5%)		
Isolated				0.003	0.957
Yes	22 (13.2%)	21 (13.4%)	43 (13.3%)		
No	145 (86.8%)	136 (86.6%)	281 (86.7%)		
Living with serenity the situation: n (%)				7.3	0.026*
Not at all	10 (5.9%)	6 (3.8%)	16 (4.9%)		
A little	36 (21.6%)	18 (11.5%)	54 (16.7%)		
A lot	121 (72.5%)	133 (84.7%)	254 (78.4%)		
Feeling confident: n (%)				2.5	0.290
Not at all	26 (15.6%)	18 (11.5%)	44 (13.6%)		
A little	57 (34.1%)	47 (29.9%)	104 (32.1%)		
A lot	84 (50.3%)	92 (58.6%)	176 (54.3%)		
Feeling pessimist: n (%)				0.3	0.853
Not at all	98 (58.7%)	94 (59.9%)	192 (59.3%)		
A little	51 (30.5%)	44 (28.0%)	95 (29.3%)		
A lot	18 (10.8%)	19 (12.1%)	37 (11.4%)		
Change the quality of sleep: n (%)				4.9	0.086
N-Miss	1	0	1		
Not at all	99 (59.6%)	112 (71.4%)	211 (65.3%)		
A little	37 (22.3%)	24 (15.3%)	61 (18.9%)		
A lot	30 (18.1%)	21 (13.4%)	51 (15.8%)		
Eating more: n (%)				11.7	0.003*
Not at all	88 (52.7%)	111 (70.7%)	199 (61.4%)		
A little	37 (22.2%)	25 (15.9%)	62 (19.1%)		
A lot	42 (25.2%)	21 (13.4%)	63 (19.4%)		
Weight changes: n (%)				8.7	0.013*
No	106 (63.5%)	121 (77.1%)	227 (70.1%)		
Loss weight	13 (7.8%)	12 (7.6%)	25 (7.7%)		
Gain weight	48 (28.7%)	24 (15.3%)	72 (22.2%)		
Smoking: n (%)				32.3	< 0.001**
Not smoke	93 (55.7%)	133 (84.7%)	226 (69.8%)		
Not at all	44 (26.3%)	18 (11.5%)	62 (19.1%)		
A little	12 (7.2%)	4 (2.5%)	16 (4.9%)		
A lot	18 (10.8%)	2 (1.3%)	20 (6.2%)		
Alcohol: n (%)				6.6	0.026*
Not drink	109 (65.3%)	116 (73.9%)	225 (69.4%)		
Not at all	44 (26.3%)	39 (24.8%)	83 (25.6%)		
A little	8 (4.8%)	1 (0.6%)	9 (2.8%)		
A lot	6 (3.6%)	1 (0.6%)	7 (2.2%)		
Drugs: n (%)				9.0	0.011*
Not use drugs	155 (92.8%)	156 (99.4%)	311 (96.0%)		
Not at all	11 (6.6%)	1 (0.6%)	12 (3.7%)		
A little	1 (0.6%)	0 (0.0%)	1 (0.3%)		
A lot	0 (0.0%)	0 (0.0%)	0 (0.0%)		
Mood medications: n (%)				0.9	0.627
Not at all	152 (91.0%)	141 (89.8%)	293 (90.4%)		
A little	9 (5.4%)	12 (7.6%)	21 (6.5%)		
A lot	6 (3.6%)	4 (2.5%)	10 (3.1%)		
Suicidal thoughts: n (%)				3.2	0.198
Not at all	156 (93.4%)	138 (87.9%)	294 (90.7%)		
A little	7 (4.2%)	14 (8.9%)	21 (6.5%)		
A lot	4 (2.4%)	5 (3.2%)	9 (2.8%)		
Psychological support: n (%)				0.3	0.586
Yes	23 (13.8%)	25 (15.9%)	48 (14.8%)		
No	144 (86.2%)	132 (84.1%)	276 (85.2%)		
Others were more scarred than the patients: n (%)				0.4	0.840
N-Miss	2	2	4		
Not at all	35 (21.2%)	37 (23.9%)	72 (22.5%)		
A little	42 (25.5%)	37 (23.9%)	79 (24.7%)		
A lot	88 (53.3%)	81 (52.3%)	169 (52.8%)		
Period of reflection: n (%)				4.5	0.104
N-Miss	0	1	1		
Not at all	40 (24.0%)	52 (33.3%)	92 (28.5%)		
A little	28 (16.8%)	29 (18.6%)	57 (17.6%)		
A lot	99 (59.3%)	75 (48.1%)	174 (53.9%)		
Covid-19 pandemic vs HIV pandemic: n (%)				–	–
Not at all	103 (62.4%)	–			
A little	30 (18.2%)	–			
A lot	32 (19.4%)	–			

Chi-Square χ^2^ Test

*p < 0.05; **p < 0.001

Regarding daily habits, a major part of the participants (65.3%) reported not to have changed the quality of sleep (59.6% of PLWH vs. 71.4% PLWOD, n.s.). Changes in nutritional supply were also analyzed: 63 patients (19.4%) referred an increase in food intake (25.2% of PLWH vs. 13.4% of PLWOD; χ^2^ = 11.7, *p* = 0.003). Notwithstanding, only the 22.2% of patients reported weight increase (28.7% of PLWH vs. 15.3% of PLWOD; χ^2^ = 8.7, *p* = 0.013). Moreover, PLWH smoked significant more than PLWOD (10.8% of PLWH vs. 1.3% of PLWOD; χ^2^ = 32.3, *p* < 0.001).

The suicidal thoughts and the self-harming ideas were also analyzed among participants. Most of the patients (90.7%) did not experience intrusive thoughts. The introspection ability among participants was analyzed and most of patients (53.9%) reported that the quarantine was a period of personal development.

Lastly, it was asked to the PLWH group if the COVID-19 may be comparable with the HIV pandemic. Most of the PLWH (62.4%) referred that the two situations were not comparable.

### Experience in Hospital Ward

PLWH have been using the hospital services for significant longer time than PLWOD (15.8 vs. 9.65 years; H = 140.2, *p* < 0.001). Furthermore, most of participants reported that the hygiene measures against COVID-19 adopted by the Hospital were adequate (95.9%) and reported to feel protected from the infection in the clinic of origin both during the previous visits (96%) and at the moment of the interview (97.2%).

Regarding relationship with healthcare providers, 247 (77.7%) patients were not afraid about the possibility that the healthcare providers may be infected by COVID-19 and could not be reference points anymore (79.8% of PLWH vs. 75.5%, n.s.). Moreover, 277 (86%) patients were not worried about the possibility that the medical staff could infect patients themselves. The majority of patients reported they would not have wanted to be informed about the sanitary situation of the ward before the visit (86.7%) and they felt supported by the healthcare providers during the COVID-19 pandemic (78.3%). Most of patients (90.3%) did not feel neglected by the sanitary system and they reported to be assisted as usual (Table S4 in the supplement materials).

### Resilience

**Fig. 1:**
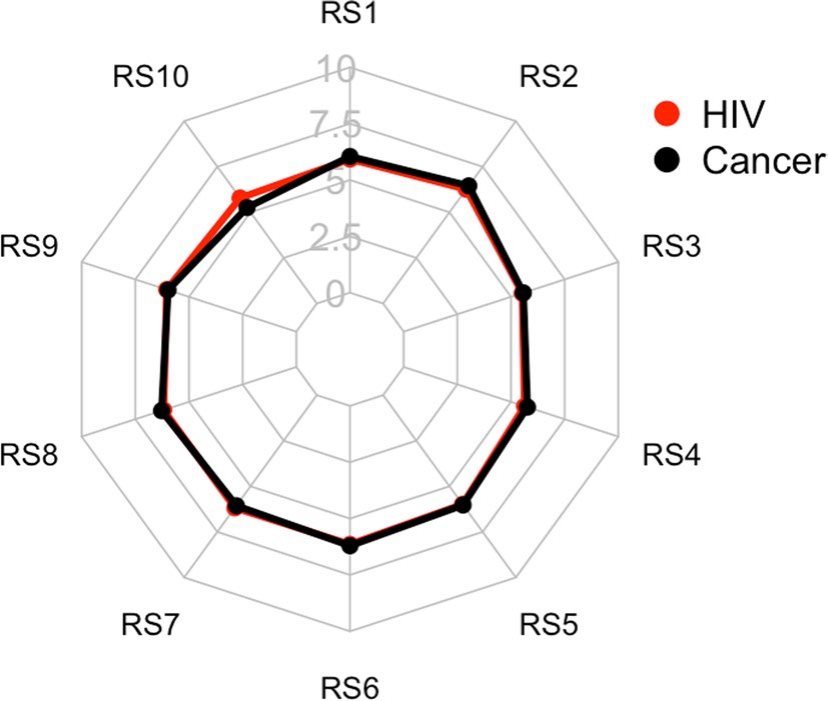
The radar plot of the means of each item in the Resilience Scale (RS). Each vertex represents each item of the RS

The resilience of the participants was investigated to study the differences between the groups in the capability to manage the critical psychological issue during the pandemic (Table S5 in the supplement materials). Participants had in general high level of resilience measured by RS (RS mean ± standard deviation: 59.7 ± 6.92). Regarding the total RS score, no significant differences were found between the groups (RS score: 59.7 in PLWH vs. 59.6 in PLWOD) except in the last item “I have enough energy to do what I should do”, in which PLWH showed higher mean scores than PLWOD (5.8 vs. 5.2, respectively; H = 9.7, *p* = 0.002) (Fig. 1, item 10).

### Linear Regression Analysis

From the original 44 variables collected through the questionnaire, factor analysis allowed to identify latent factors defined based on the loading (Figure S1) as feeling of anger, need of attention, concern of the infection, psychological problems, anxiety/concern, coping and lockdown conditions. All the factors were included in the linear regression to test their effect on RS.

No significant differences were found between the groups in the Resilience Score (R^2^ = − 1.0, *p* = 0.410). However, who was unemployed [R^2^ = − 3.52; 95% CI (− 6.68 − 0.35); *p* = 0.029], who showed more anger feelings [R^2^ = − 1.76; 95% CI (− 2.67 − 0.85); *p* < 0.001], who showed more anxiety/concern [R^2^ = − 1.56; 95% CI (− 2.30 − 0.82); *p* < 0.001] and who had a history of psychological problems during the lockdown [R^2^ = − 2.18; 95% CI (− 3.17 − 1.18); *p* < 0.001] displayed a lower level of resilience regardless of chronic disease (Table S6 in the supplement materials).

## Discussion

This study evaluates the emotional experiences, the psychological effects, and the resilience among PLWCD, comparing PLWH to PLWOD, during the pandemic of SARS-CoV-2 in a centre of North Italy.

Here, unlike previous studies [16, 41], in general we found that pandemic has not modified the psychological well-being of the participants. Patients in general underestimated the risk of belonging to a frail category and the majority of responders were optimistic about the evolution of the emergency, also with the hope of vaccination. However, PLWOD were significant more calm, less troubled, and more serene than PLWH, despite oncological patients were more worried than PLWH about the clinical outcomes of the COVID-19 infection. In fact, PLWH sustained that the ART was a protective factor for the infection, as initially published [40]. The sense of loneliness and isolation were mitigated by the possibility of using the new technologies [42], such as smartphone and online platforms of meeting. Noteworthy, a greater number of PLWH than PLWOD lived alone during the quarantine, but this aspect did not change the outcomes. In general, all the participants referred to be supported by the healthcare providers, put their trust in them and felt safe to go to the Hospital for their visits: hygienic measures and organization were adequate. These aspects probably allowed to maintain the adherence to the therapies and to the continuum of care, contrary to what was found in a previous study in HIV patients [41]. Our result may be explained by the fact that the care continuum in the Hospital during the lockdown was also guaranteed through telemedicine, which was a useful tool to preserve the care involvement [43]. We also explored the parallelisms between the COVID-19 and HIV pandemics and patients reported that the stigma due to the HIV positivity was not comparable at all with the COVID-19 positivity.

The quarantine did not upset drastically the habits of the participants, unlike a previous study [16]. Our results suggest that PLWH [16] and PLWOD were able to adapt themselves and their behaviours to the situation, indeed patients continued to take pleasure from their daily activities as usual and active coping strategies, such as passing time with new activities. These aspects were protective factors for the management of the chronic diseases and played an important role in the outcome of the patient’s burden [20]. Some participants (PLWH > PLWOD) referred to eat more and therefore to gain weight, this is maybe linked to the reduction of physical activity and to the increase of cooking activities. Few differences in the habits were detected between the groups: PLWH drank alcohol and smoked cigarettes more than PLWOD, slightly in contrast to other studies [16]. It is important to consider that for PLWOD could be very difficult to differentiate the effects in the daily habits due to the therapies and due to COVID-19. Lastly, patients kept informed (PLWOD > PLWH) about the COVID-19 disease and understood the importance of the individual protection, such as facial masks and physical distancing, and paid attention to the COVID-19 symptoms. The period of the quarantine was an opportunity of reflection. Also, patients reported that the information obtained from the health care providers were preferred than the politicians’ ones, in line with others [16].

Patients showed high level of resilience, with a ceiling effect in the RS score, and no significant differences were found between groups. Unlike a previous study [41], the participants’ age did not influence the resilience score. In our study emerged that those who lived in a disadvantaged situation, those who had a history of psychological disorders and patients that felt negative emotions, such as angry, anxiety and concern, during the lockdown had lower values in the RS independently by the group.

Our study has some strength. To our knowledge, first: little is known about the emotional experience and the psychological effects of frailty patients that lived with an oncological disease during the pandemic and, second: we compared the emotional and psychological outcomes due to the pandemic in two different frailty chronic population. The study describes the importance of positive psychology in the management of the critical situations and sheds a new light on resilience and coping strategies during acute crisis in patients that live with the chronicity. In particular, the presence of adaptive coping and high level of resilience in PLWCD could reduce drastically the burden due to the pandemic.

Some limitations need to be acknowledged. First, our work does not guarantee the generalisability of the results because the Italian situation cannot be compared to the others, in particular the work was conducted in a single centre included in service-rich city and with a free national health and welfare system. Second, we did not include a control group composed of healthy people. Additionally, the questionnaires were conducted in presence of a healthcare provider which raises potential response bias in the interviewed. Moreover, we did not collect data on disease stage in cancer patients. Since the majority of PLWOD was following a day-hospital care regime for prevention of recurrence, the underestimation of belonging to a frail category in PLWOD could be linked with the evolution of the disease. Last concern is the difference in the demographic characteristics and cultural factors [4] between the groups, such as the different number of foreigner people and age differences.

## Conclusions

Generalizing, in our study patients’ perception is that the pandemic did not have significant psychological effects. However, we found that PLWOD were more calm, less troubled, and more serene than PLWH. Few changes in the daily habits in the PLWH were found compared to PLWOD and PLWH showed higher distress than PLWOD according to comparable resilience score. High levels of social support and resilience were recorded in both groups. Last, patients with psychological problems, feelings of anger and concern deserve more attention as they show lower levels of resilience. Further investigations are needed to confirm this finding and to compare PLWCD with the general population.

## Supplementary Information

Below is the link to the electronic supplementary material.

Supplementary file1 (DOC 101 KB)

Supplementary file2 (DOCX 155 KB)

## Data Availability

Dataset generated and analysed during the current study are available in the Google Drive repository. https://docs.google.com/spreadsheets/d/1vPAeR2CRaQXFmcK_oB23MsRY1o9AkzTB6HLeVyTbX8c/edit?usp=sharing
