# Supplementary material for: Psychological and Emotional Impact of COVID-19 Pandemic on People Living with Chronic Disease: HIV and Cancer
Source: AIDS Behav. 2022 Mar 6;26(9):2920–30. doi: 10.1007/s10461-022-03638-0 (PMC8898333; doi:10.1007/s10461-022-03638-0)
Supplement: Supplementary file 1 — Supplementary file1 (DOC 101 KB) [file 10461_2022_3638_MOESM1_ESM.doc]

**QUESTIONARIO PER LA RILEVAZIONE DEI VISSUTI DEL PAZIENTI NEL CORSO DELL’EPIDEMIA DA COVID 19**

**Reparto _________________________**

1.**DATI ANAGRAFICI**

**1.Sesso**

1[] F

2[] M

3[] Altro: **_________________________ (**specificare)

**2.Età:**

**_________** (anni)

1[] <30

2[] 30-45

3[] 45-60

4[] >60

**3.Nazionalità**

1[] Italiano

2[] Straniero

Se straniero indicare la nazionalità __________________________________________________

**4.Livello di istruzione**

1[] licenza elementare

2[] licenza media

3[] diploma

4[] laurea

**5.Professione attuale**

1[] occupato

Che tipo ____________________________________

2 [] disoccupato

Da quanto tempo _____________________________

3 [] casalinga

4 [] pensionato

5 [] studente

6 [] altro ________________________________

2.**RETE FAMILIARE**

**1.Stato di convivenza attuale** (più risposte possibili)

1[] da solo

2[] moglie/ marito,

3[] genitore/i

4[] figli,

5[] fratelli/ sorelle,

6[] nonni

7[] nipoti

8[] altro

**2.Con chi viveva prima dell’emergenza Covid?** (possibili più risposte)

1[] da solo

2[] moglie/ marito,

3[] genitore/i

4[] figli,

5[] fratelli/ sorelle,

6[] nonni

7[] nipoti

8[] altro

**3.Con chi ha vissuto durante la fase più acuta dell’emergenza Covid?** (possibili più risposte)

1[] da solo

2[] moglie/ marito,

3[] genitore/i

4[] figli

5[] fratelli/ sorelle,

6[] nonni,

7[] nipoti,

8[] altro

**4.Quanto le persone che ha indicato solitamente l’aiutano:**

*(segnare il pallino corrispondete al grado di aiuto e al/ai parente/i espressi in precedenza)*

|  | Moglie/marito | Genitore/i | Figli | Fratelli/sorelle | Nonni | Nipoti | altro |
| --- | --- | --- | --- | --- | --- | --- | --- |
| Per nulla |  |  |  |  |  |  |  |
| Poco |  |  |  |  |  |  |  |
| Molto |  |  |  |  |  |  |  |

**5.Nel periodo di emergenza Covid in che tipo di abitazione risiedeva?**

1[] Monolocale,

2[] bilocale,

3[] trilocale,

4[] abitazione più ampia, con balcone o terrazzo/con giardino

3.**INFORMAZIONI LEGATE ALLA PATOLOGIA**

**1.Da che tipo di patologia è affetto?**

1[] Malattia oncologica

2[] HIV

3[] Altro: **_________________________ (**specificare)

**2.Da quanto tempo le è stata diagnosticata?**

1[] Da meno di 5 anni

2[] Da più di 5 anni

**3.Ha mai sofferto di problemi psicologici?**

1[] Sì

2[] No

**4.Se sì quali?** (possibili più risposte)

1[] disturbi d’ansia,

2[] disturbi di depressione,

3[] altri problemi psicologici

Specificare ______________________________________________________________________

**5.Quando?**

________________________________________________________________________________________________________________________________________________________________________________________________________________________________________________

4.**VISSUTO EMOTIVO E STRATEGIE DI COPING**

**1.In riferimento all’emergenza sanitaria Covid, in quel periodo ritiene di essersi sentito:** (possibili più risposte)

1[] tranquillo,

2[] indifferente,

3[] triste,

4[] in colpa nei confronti dei familiari,

5[] impotente,

6[] arrabbiato,

7[] nervoso

8[] irrequieto,

9[] preoccupato,

10[] in ansia,

11[] spaventato,

12[] in preda al panico

**2.Si è sentito in grado di gestire serenamente la situazione?**

1[] per nulla

2[] poco

4[] molto

**3.Pensa che le indicazioni fornite dai medici siano state utili?**

1[] per nulla

2[] poco

4[] molto

**4.** **Pensa che le indicazioni fornite dal ministro della sanità siano state utili?**

1[] per nulla

2[] poco

4[] molto

**5.Ha cercato di prestare molta attenzione ai possibili segnali di malessere fisico riconducibili al coronavirus?**

1[] per nulla

2[] poco

4[] molto

**6.È stato preoccupato per problemi economici legati all’emergenza Covid?**

1[] per nulla

2[] poco

4[] molto

**7.Si è sentito più solo del solito?**

1[] per nulla

2[] poco

4[] molto

**8.Per gestire le sue emozioni e lo stress, ha usato esercizi di rilassamento o tecniche meditative?**

1[] per nulla

2[] poco

4[] molto

**9.Ha continuato a ricavare piacere dalle cose che faceva?**

1[] per nulla

2[] poco

4[] molto

**10.Nonostante le limitazioni imposte dall’emergenza Covid, è riuscito a mantenere buone relazioni con gli altri?**

1[] per nulla

2[] poco

4[] molto

**11.È riuscito a manifestare i suoi pensieri e le sue emozioni ai suoi familiari, rispetto al vissuto Covid?**

1[] per nulla

2[] poco

4[] molto

**12.Si è tenuto in contatto telefonicamente con altri pazienti con la sua stessa patologia?**

1[] per nulla

2[] poco

4[] molto

**13.La qualità del suo sonno e del suo riposo è cambiata?**

1[] per nulla

2[] poco

4[] molto

**14.Nel periodo segnato dall’emergenza Covid, si è sentito pessimista/senza speranza?**

1[] per nulla

2[] poco

4[] molto

**15.Si è sentito fiducioso e ottimista?**

1[] per nulla

2[] poco

4[] molto

**16.Per gestire le sue emozioni ha mangiato più del solito?**

1[] per nulla

2[] poco

4[] molto

**17. Lei fuma sigarette?**

1[] si

2[] no

**18.Ha fumato più del solito?**

1[] per nulla

2[] poco

4[] molto

**19. Lei abitualmente beve alcolici?**

1[] si

2[] no

**20.Ha bevuto più alcolici?**

1[] per nulla

2[] poco

4[] molto

**21. Lei abitualmente fa uso di sostanze stupefacenti?**

1[] si

2[] no

**22. Ha fatto uso di sostanze stupefacenti più del solito?**

1[] per nulla

2[] poco

4[] molto

**23.Ha utilizzato più farmaci per gestire il suo umore e il suo stato d’ansia?**

1[] per nulla

2[] poco

4[] molto

**24.Ha pensato che sarebbe stato meglio non esserci più?**

1[] per nulla

2[] poco

4[] molto

**25.Si è sentito aiutato dalla fede in questo periodo?**

1[] per nulla

2[] poco

4[] molto

**26.Ha sentito il bisogno di parlare con uno psicologo?**

1[] Si

2[] No

**27.Secondo il suo parere, gli altri in genere hanno avuto più paura di Lei riguardo al coronavirus?**

1[] per nulla

2[] poco

4[] molto

**28.Ritiene che l’esperienza di questo periodo possa rappresentare per lei un’occasione di crescita come persona?**

1[] per nulla

2[] poco

4[] molto

**29.** **Nel periodo segnato dall’emergenza Covid, ha subito variazioni di peso?**

1[] no

2[] ho perso peso

3[] sono aumentato di peso

5.**ESPERIENZA COVID NEL REPARTO DI APPARTENENZA**

**1.Da quanto tempo usufruisce dei servizi dell’ospedale per la sua patologia?** (anni)

**_________**

**2.Durante l’emergenza Covid ha proseguito il trattamento terapeutico per la sua patologia?**

1[] sì

2[] no

**3.Se sì, in che regime è stato curato?**

1[] ricovero

2[] day hospital

**4.Quanto ha pensato di essere più a rischio di contrarre il Covid in quanto immunodepresso?** 1[] per nulla

2[] poco

4[] molto

**5.Quanto ha temuto che l’infezione da Covid potesse peggiorare la sua malattia mettendola a rischio di morte?**

1[] per nulla

2[] poco

4[] molto

**6.Secondo lei, nel suo reparto, la gestione sanitaria legata al Covid è stata attuata adeguatamente (misure di protezione, sanificazione, dispositivi di sicurezza, rispetto del distanziamento sociale, ecc…)?**

1[] Sì

2[] No

**7.Se no, perché?**

________________________________________________________________________________________________________________________________________________________________

**8.Durante la fase acuta dell’emergenza COVID, nel suo reparto, si è sentito protetto dal rischio di contrarre il Covid?**

1[] Sì

2[] No

**9.Se no, perché?**

________________________________________________________________________________________________________________________________________________________________

**10.Oggi, nel suo reparto, si sente protetto dal rischio di contrarre il Covid?**

1[] Sì

2[] No

**11.Se no perché?**

________________________________________________________________________________________________________________________________________________________________

**12.È stato/a tentato/a di chiedere di interrompere la cura a causa del covid?**

1[] si

2[] no

**13.Durante la fase acuta dell’emergenza Covid, ha scelto di disdire l’appuntamento/prelievo per paura del contagio da Covid?**

1[] si

2[] no

**14.Ha pensato di essere stato/a contagiato/a dal coronavirus?**

1[] Sì

2[] No

**15.Se sì, quanto si è preoccupato/a?**

1[] per nulla

2[] poco

4[] molto

**16.E’ stato preoccupato dal fatto che i medici del suo reparto potessero aver contratto il Covid e quindi non potessero essere più i suoi riferimenti in questo periodo?**

1[] per nulla

2[] poco

4[] molto

**17. L’ha preoccupata il pensiero che i medici del suo reparto potessero aver contratto il Covid e potessero contagiarla?**

1[] per nulla

2[] poco

4[] molto

**18.Avrebbe gradito essere informato sulla situazione sanitaria del reparto prima di accedervi per l’appuntamento programmato?**

1[] Sì

2[] No

**19.Ha avuto timore che l’emergenza Covid potesse diminuire l’attenzione nei confronti della sua patologia e quindi dell’adeguata assistenza sanitaria a lei solitamente dedicata?**

1[] per nulla

2[] poco

4[] molto

***Solo per pz HIV***

**20.L’infezione da Covid, in base alle conseguenze cliniche, sociali e relazionali che comporta, mi ha ricordato i vissuti legati all’aver contratto infezione da HIV?**

1[] mai

2[] poco

4[] molto

**21.Se sì, in che modo? (più risposte possibili)**

1[] paura di essere isolato dalle altre persone,

2[] paura di essere pericoloso per gli altri,

3[] altro

**22.Si è sentito supportato emotivamente dai suoi medici di riferimento in reparto durante il covid?**

1[] per nulla

2[] poco

4[] molto

**23.Si è sentito trascurato dal punto di vista sanitario in questo periodo?**

1[] per nulla

2[] poco

4[] molto

**24.Si è sentito assistito come al solito dai suoi medici di reparto?**

1[] per nulla

2[] poco

4[] molto

**25. Ritiene che indossare solo la mascherina è sufficiente a proteggerla dal contagio di covid?**

1[] per nulla

2[] poco

4[] molto

**26.Nel caso in cui fosse disponibile un vaccino anti-COVID vorrebbe effettuarlo:**

1[] non appena fosse possibile

2[] aspetterei qualche tempo per vedere l’effetto sulle persone che lo hanno fatto

3[] non vorrei mai farlo

**27. Ha mai fatto visite in telemedicina?**

[] si

[] no

**28.In base alla sua esperienza, ritiene che le visite fatte in telemedicina siano equivalenti a quelle ambulatoriali fatte di persona?**

1[] per nulla

2[] poco

4[] molto

28.2 In base alla sua esperienza, farebbe delle visite in telemedicina anche quando l’emergenza sanitaria sarà finita?

1[] si

2[] no

28.2.1 Se NO, perché non la rifarebbe?

________________________________________________________________________________

________________________________________________________________________________

28.3 Se SI, quanto questi motivi potrebbero incidere sulla scelta di svolgere visite in telemedicina al di fuori dell’emergenza sanitaria:

|  | per nulla | poco | molto |
| --- | --- | --- | --- |
| risparmio di tempo |  |  |  |
| evitare permessi al lavoro |  |  |  |
| tutela della privacy |  |  |  |
| evitare di spostarsi da casa se si abita lontano |  |  |  |

28.3.1 Ci sono altri motivi che potrebbero incidere sulla scelta di svolgere visite in telemedicina al di fuori dell’emergenza sanitaria? (se no, lasciare in bianco)

________________________________

**29. Ritiene che l’esperienza di questo periodo possa rappresentare (per la Sanità, l’ospedale e i suoi curanti) un’occasione di miglioramento nelle cure e nell’approccio al paziente?**

1[] per nulla

2[] poco

4[] molto

6.**INFORMAZIONI SULL’INFEZIONE DA COVID19**

**1.Si è tenuto informato/a sull’andamento dell’epidemia?**

1[] Si

2[] No

**2.Se si: con quale frequenza**

1 [] ogni ora

2 [] ogni 3-5 ore

3 [] una volta al giorno

4 [] meno di una volta al giorno

**3.Quale fonte principale di informazione ha utilizzato? (più risposte possibili)**

1 []media tradizionali (stampa, televisione)

2 [] siti internet(Google, siti Web, quotidiani online)

3 [] social media(Facebook, Instagram, YouTube)

4 [] app di messaggistica (WhatsApp, Telegram, SMS)

5 [] personale sanitario

6 [] familiari

7 [] amici

8 [] altro

**4.Secondo Lei l’inquinamento ambientale ha influenzato la comparsa e la diffusione del COVID?**

1 [] si

2 [] no

3 [] non so

**5.Secondo Lei il sovraffollamento globale ha influenzato la comparsa e la diffusione del COVID?**

1 [] si

2 [] no

3 [] non so

**6.Secondo Lei l’immigrazione ha influenzato la comparsa e la diffusione del COVID?**

1 [] si

2 [] no

3 [] non so

**7.Secondo Lei questo coronavirus è dovuto a:**

1 [] evento naturale

2 [] manipolazione da parte di qualcuno

3 [] non so

**Resilience Scale (RS) (Wagnild & Young)**

Pensando a te stesso, barra la casella che meglio rappresenta il tuo grado di accordo con le affermazioni che seguono, usando la scala sotto riportata. Ricorda che non ci sono risposte giuste o sbagliate. Scegli la risposta che meglio descrive il tuo modo di essere o di comportarti.

|  | Fortemente in disaccordo | In disaccordo | Abbastanza in disaccordo | Né in accordo né in disaccordo | Abbastanza d’accordo | D’accordo | Fortemente d’accordo |
| --- | --- | --- | --- | --- | --- | --- | --- |
| Di solito, in un modo o nell’altro, riesco a cavarmela | 1 | 2 | 3 | 4 | 5 | 6 | 7 |
| L’aver portato a termine qualcosa nella mia vita mi rende orgoglioso | 1 | 2 | 3 | 4 | 5 | 6 | 7 |
| Normalmente accetto quanto mi riserva il destino | 1 | 2 | 3 | 4 | 5 | 6 | 7 |
| Sono amico di me stesso | 1 | 2 | 3 | 4 | 5 | 6 | 7 |
| Sono determinato | 1 | 2 | 3 | 4 | 5 | 6 | 7 |
| Mantengo vivo il mio interesse per quanto mi circonda | 1 | 2 | 3 | 4 | 5 | 6 | 7 |
| Credere in me stesso mi aiuta a superare i tempi duri | 1 | 2 | 3 | 4 | 5 | 6 | 7 |
| La mia vita ha un senso | 1 | 2 | 3 | 4 | 5 | 6 | 7 |
| Quando sono in una situazione difficile di solito riesco a trovare una soluzione | 1 | 2 | 3 | 4 | 5 | 6 | 7 |
| Ho sufficienti energie per fare ciò che devo fare | 1 | 2 | 3 | 4 | 5 | 6 | 7 |
