# Supplementary material for: Psychological and Emotional Impact of COVID-19 Pandemic on People Living with Chronic Disease: HIV and Cancer
Source: AIDS Behav. 2022 Mar 6;26(9):2920–30. doi: 10.1007/s10461-022-03638-0 (PMC8898333; doi:10.1007/s10461-022-03638-0)
Supplement: Supplementary file 2 — Supplementary file2 (DOCX 155 KB) [file 10461_2022_3638_MOESM2_ESM.docx]

**Psychological and emotional impact of COVID-19 pandemic on people living with chronic disease: HIV and cancer.**

Figure 2: The figure showed the main results of the study.

**Authors:** Emanuele Focà^1§^, Chiara Fornari^1§^, Stefania Arsuffi^1^, Maria Chiara Vetrano^1^, Stefano Calza^2^, Stefano Renzetti^2^, Silvia Copeta^3^, Alfredo Berruti^3^, Francesco Castelli^1^, Silvia Compostella^4#^, Eugenia Quiros-Roldan^1#^

**Affiliations:**

1: University Department of Infectious and Tropical Diseases, University of Brescia and ASST Spedali Civili Hospital, Brescia, Italy

2: Unit of Biostatistics and Bioinformatics, Department of Molecular and Translational Medicine, University of Brescia, Brescia, Italy

3: Department of Medical and Surgical Specialties, Radiological Sciences, and Public Health, Medical Oncology, University of Brescia at ASST Spedali Civili, Brescia, Italy.

4: Unit of Infectious Diseases of ASST Spedali Civili Hospital, Brescia, Italy

**Corresponding to:**

Emanuele Focà MD, PhD

Unit of Infectious and Tropical Diseases, Department of Clinical and Experimental Sciences, University of Brescia and ASST Spedali Civili, Brescia, Italy

[emanuele.foca@unibs.it](mailto:emanuele.foca@unibs.it)

**Supplementary Materials**

| TABLE S1. Pathological history | | | | | |
| --- | --- | --- | --- | --- | --- |
| Characteristics | **Observed** | | **Total (N=324)** | **χ^2^ Test** | **p- value** |
|  | PLWH (N=167) | PLWOD (N=157) |  |  |  |
| History of psychological problems: n (%) | | |  | 1.2 | .268 |
| Yes | 45 (26.9%) | 34 (21.7%) | 79 (24.4%) |  |  |
| No | 122 (73.1%) | 123 (78.3%) | 245 (75.6%) |  |  |
| If positive history of psychological problems. Psychological disorders: n (%)^a^ | | | | 1.2 | .543 |
| Anxiety | 20 (29.4%) | 26 (37.7%) | 46 (33.6%) |  |  |
| Depression | 41 (60.3%) | 38 (55.1%) | 79 (57.7%) |  |  |
| Others | 7 (10.3%) | 5 (7.2%) | 12 (8.7%) |  |  |
| Adherence to the therapy during the pandemic: n (%) | | |  | 0.04 | .842 |
| N-Miss | 1 | 42 | 43 |  |  |
| Yes | 161 (97.0%) | 112 (97.4%) | 273 (97.2%) |  |  |
| No | 5 (3.0%) | 3 (2.6%) | 8 (2.8%) |  |  |
| Care regime: n (%) | | |  | 10.4 | .001* |
| N-Miss | 20 | 9 | 29 |  |  |
| Hospitalization | 3 (2.0%) | 17 (11.5%) | 20 (6.8%) |  |  |
| Day Hospital | 144 (98.0%) | 131 (88.5%) | 275 (93.2%) |  |  |
| Ask to stop the therapy: n (%) | | |  | 0.2 | .692 |
| N-Miss | 2 | 24 | 26 |  |  |
| Yes | 2 (1.2%) | 1 (0.8%) | 3 (1.0%) |  |  |
| No | 163 (98.8%) | 132 (99.2%) | 295 (99.0%) |  |  |
| Cancel an appointment: n (%) | | |  | 1.6 | .204 |
| N-Miss | 1 | 9 | 10 |  |  |
| Yes | 6 (3.61%) | 2 (1.35%) | 8 (2.5%) |  |  |
| No | 160 (96.4%) | 146 (98.6%) | 306 (97.5%) |  |  |
| Thought to be infected and concern about the infection: n (%) | | | | 1.3 | .003* |
| No | 116 (69.5%) | 135 (86.0%) | 251 (77.5%) |  |  |
| Not at all | 21 (12.6%) | 6 (3.8%) | 27 (8.3%) |  |  |
| A little | 15 (9.0%) | 8 (5.1%) | 21 (7.1%) |  |  |
| A lot | 15 (9.0%) | 8 (5.1%) | 21 (7.1%) |  |  |
| Note: ^a^ more than one answer option was allowed (N=137; total PLWH: 68; total PLWOD: 69). Chi-Square χ^2^ Test. * p < .05; ** p < .001 | | | | | |

| TABLE S2. Information about COVID-19 | | | | | |
| --- | --- | --- | --- | --- | --- |
| Characteristics | **Observed** | | **Total (N=324)** | **χ^2^ Test** | **p- value** |
|  | PLWH (N=167) | PLWOD (N=157) |  |  |  |
| Source of information: n (%)^a^ | | |  | 48.3 | .325 |
| Television | 147 (35.0%) | 144 (43.4%) | 291 (38.7%) |  |  |
| Internet | 121 (28.8%) | 91 (27.4%) | 212 (28.2%) |  |  |
| Social media | 71 (16.9%) | 47 (14.2%) | 118 (15.7%) |  |  |
| Messaging app | 5 (1.2%) | 3 (0.9%) | 8 (1.1%) |  |  |
| Family | 26 (6.2%) | 16 (4.8%) | 42 (5.6%) |  |  |
| Healthcare providers | 30 (7.1%) | 22 (6.6%) | 52 (6.9%) |  |  |
| Friends | 18 (4.3%) | 9 (2.7%) | 27 (3.6%) |  |  |
| Others | 2 (0.5%) | 0 (0.0%) | 2 (0.3%) |  |  |
| Frequency of the access to the information: n (%) | | |  | 7.5 | .013* |
| No | 8 (4.8%) | 1 (0.6%) | 9 (2.8%) |  |  |
| Each hour | 11 (6.6%) | 14 (9.0%) | 25 (7.7%) |  |  |
| Every 3-5 hours | 71 (42.5%) | 90 (57.3%) | 161 (49.7%) |  |  |
| Once a day | 64 (38.3%) | 44 (28.0%) | 108 (33.3%) |  |  |
| Less than once a day | 13 (7.8%) | 8 (5.1%) | 21 (6.5%) |  |  |
| Information from medical staff: n (%) | | |  | 2.6 | .273 |
| N-Miss | 36 | 25 | 61 |  |  |
| Not at all | 14 (10.7%) | 14 (10.6%) | 28 (10.6%) |  |  |
| A little | 18 (13.7%) | 28 (21.2%) | 46 (17.5%) |  |  |
| A lot | 99 (75.6%) | 90 (68.2%) | 189 (71.9%) |  |  |
| Information from politicians: n (%) | | |  | 0.9 | .635 |
| N-Miss | 2 | 1 | 3 |  |  |
| Not at all | 39 (23.6%) | 41 (26.3%) | 80 (24.9%) |  |  |
| A little | 66 (40.0%) | 66 (42.3%) | 132 (41.1%) |  |  |
| A lot | 60 (36.4%) | 49 (31.4%) | 109 (34.0%) |  |  |
| Elements that influenced the Covid-19 pandemic: n (%) | | |  |  |  |
| Environmental pollution |  |  |  | 0.6 | .747 |
| Yes | 92 (55.1%) | 80 (51.0%) | 172 (53.1%) |  |  |
| No | 36 (21.6%) | 38 (24.2%) | 74 (22.8%) |  |  |
| I do not know | 39 (23.4%) | 39 (24.8%) | 78 (24.1%) |  |  |
| Global overcrowding |  |  |  | 1.7 | .432 |
| N-Miss | 1 | 0 | 1 |  |  |
| Yes | 120 (72.3%) | 122 (77.7%) | 242 (74.9%) |  |  |
| No | 27 (16.3%) | 18 (11.5%) | 45 (13.9%) |  |  |
| I do not know | 19 (11.4%) | 17 (10.8%) | 36 (11.1%) |  |  |
| Immigration |  |  |  | 2.8 | .241 |
| Yes | 95 (56.9%) | 97 (61.9%) | 192 (59.3%) |  |  |
| No | 65 (38.9%) | 49 (31.2%) | 114 (35.2%) |  |  |
| I do not know | 7 (4.2%) | 11 (7.0%) | 18 (5.6%) |  |  |
| Source of Covid-19 pandemic: n (%) | | |  | 2.1 | .335 |
| Natural element | 35 (21.0%) | 39 (24.8%) | 74 (22.8%) |  |  |
| Manipulation | 91 (54.5%) | 73 (46.5%) | 164 (50.6%) |  |  |
| I do not know | 41 (24.6%) | 45 (28.7%) | 86 (26.5%) |  |  |
| Note: ^a^ more than one answer option was allowed (N=752; total PLWH: 420; total PLWOD: 332). Chi-Square χ^2^ Test. * p < .05; ** p < .001 | | | | | |

| TABLE S3. Social support and coping strategies | | | | | |
| --- | --- | --- | --- | --- | --- |
| Characteristics | **Observed** | | **Total (N=324)** | **χ^2^ Test** | **p- value** |
|  | PLWH (N=167) | PLWOD (N=157) |  |  |  |
| Family support in everyday life: n (%) | | |  | 0.02 | .992 |
| N-Miss | 40 | 18 | 58 |  |  |
| Not at all | 7 (5.3%) | 8 (5.7%) | 15 (5.6%) |  |  |
| A little | 17 (13.5%) | 18 (13.0%) | 35 (13.2%) |  |  |
| A lot | 103 (81.2%) | 113 (81.3%) | 216 (81.2%) |  |  |
| Loneliness during the quarantine: n (%) | | |  | 4.1 | .130 |
| Not at all | 101 (60.5%) | 100 (63.7%) | 201 (62.0%) |  |  |
| A little | 29 (17.4%) | 35 (22.3%) | 64 (19.8%) |  |  |
| A lot | 37 (22.2%) | 22 (14.0%) | 59 (18.2%) |  |  |
| Maintenance of good relationship with others: n (%) | | |  | 5.3 | .069 |
| Not at all | 4 (2.4%) | 3 (1.9%) | 7 (2.2%) |  |  |
| A little | 22 (13.2%) | 9 (5.7%) | 31 (9.6%) |  |  |
| A lot | 141 (84.4%) | 145 (92.4%) | 286 (88.3%) |  |  |
| Express thoughts and concerns to the family: n (%) | | |  | 0.4 | .814 |
| N-Miss | 2 | 4 | 6 |  |  |
| Not at all | 13 (7.9%) | 15 (9.8%) | 28 (8.8%) |  |  |
| A little | 20 (12.1%) | 17 (11.1%) | 37 (11.6%) |  |  |
| A lot | 132 (80.0%) | 121 (79.1%) | 253 (79.6%) |  |  |
| Telephone contact with other patients: n (%) | | |  | 8.3 | .016* |
| N-Miss | 0 | 6 | 6 |  |  |
| Not at all | 118 (70.7%) | 116 (76.8%) | 234 (73.6%) |  |  |
| A little | 20 (12.0%) | 5 (3.3%) | 25 (7.9%) |  |  |
| A lot | 29 (17.4%) | 30 (19.9%) | 59 (18.6%) |  |  |
| Support given by faith: n (%) | | |  | 19.0 | < .001** |
| N-Miss | 1 | 4 | 5 |  |  |
| Not at all | 102 (61.4%) | 62 (40.5%) | 164 (51.4%) |  |  |
| A little | 32 (19.3%) | 29 (19.0%) | 61 (19.1%) |  |  |
| A lot | 32 (19.3%) | 62 (40.5%) | 94 (29.5%) |  |  |
| Use of meditation techniques: n (%) | | |  | 3.3 | .189 |
| N-Miss | 0 | 1 | 1 |  |  |
| Not at all | 128 (76.6%) | 132 (84.6%) | 260 (80.5%) |  |  |
| A little | 16 (9.6%) | 9 (5.8%) | 25 (7.7%) |  |  |
| A lot | 23 (13.8%) | 15 (9.6%) | 38 (11.8%) |  |  |
| Take pleasure to the activities: n (%) | | |  | 1.3 | .511 |
| Not at all | 16 (9.58%) | 15 (9.55%) | 31 (9.6%) |  |  |
| A little | 36 (21.6%) | 26 (16.6%) | 62 (19.1%) |  |  |
| A lot | 115 (68.9%) | 116 (73.9%) | 231 (71.3%) |  |  |
| Note: Chi-Square χ^2^ Test. * p < .05; ** p < .001 | | | | | |

| TABLE S4. Experience in the hospital ward | | | | | | |
| --- | --- | --- | --- | --- | --- | --- |
| Characteristics | **Observed** | | **Total (N=324)** | **Kruskall-Wallis H Test** | **χ^2^ Test** | **p- value** |
|  | PLWH (N=167) | PLWOD (N=157) |  |  |  |  |
| Time of use, yr: mean (sd) | 15.8 (9.5) | 9.65 (4.5) | 9.9 (9.5) | 140.2 |  | < .001** |
| Hygiene measures were adequate: n (%) | | |  |  | 0.06 | .813 |
| N-Miss | 6 | 0 | 6 |  |  |  |
| Yes | 154 (95.7%) | 151 (96.2%) | 305 (95.9%) |  |  |  |
| No | 7 (4.3%) | 6 (3.8%) | 13 (4.1%) |  |  |  |
| Feeling protect during the previous visits: n (%) | | |  |  | 0.2 | .689 |
| N-Miss | 10 | 16 | 26 |  |  |  |
| Yes | 150 (95.5%) | 136 (96.5%) | 286 (96.0%) |  |  |  |
| No | 7 (4.5%) | 5 (3.5%) | 12 (4.0%) |  |  |  |
| Feeling protect during the current visit: n (%) | | |  |  | 0.2 | .672 |
| N-Miss | 1 | 0 | 1 |  |  |  |
| Yes | 162 (97.6%) | 152 (96.8%) | 314 (97.2%) |  |  |  |
| No | 4 (2.4%) | 5 (3.2%) | 9 (2.8%) |  |  |  |
| Healthcare provider as a point: n (%) | | |  |  | 0.9 | .653 |
| N-Miss | 4 | 2 | 6 |  |  |  |
| Not at all | 130 (79.8%) | 117 (75.5%) | 247 (77.7%) |  |  |  |
| A little | 16 (9.8%) | 19 (12.3%) | 35 (11.0%) |  |  |  |
| A lot | 17 (10.4%) | 19 (12.3%) | 36 (11.3%) |  |  |  |
| Concern of being infected by the medical staff: n (%) | | |  |  | 0.05 | .976 |
| N-Miss | 1 | 1 | 2 |  |  |  |
| Not at all | 143 (86.2%) | 134 (85.9%) | 277 (86.0%) |  |  |  |
| A little | 15 (9.0%) | 15 (9.6%) | 30 (9.3%) |  |  |  |
| A lot | 8 (4.8%) | 7 (4.5%) | 15 (4.7%) |  |  |  |
| To be informed about the sanitary situation: n (%) | | |  |  | 0.4 | .547 |
| Yes | 24 (14.4%) | 19 (12.1%) | 43 (13.3%) |  |  |  |
| No | 143 (85.6%) | 138 (87.9%) | 281 (86.7%) |  |  |  |
| Emotional support: n (%) | | |  |  | 7.3 | .026* |
| N-Miss | 38 | 10 | 48 |  |  |  |
| Not at all | 21 (16.3%) | 11 (7.5%) | 32 (11.6%) |  |  |  |
| A little | 16 (12.4%) | 12 (8.2%) | 28 (10.1%) |  |  |  |
| A lot | 92 (71.3%) | 124 (84.3%) | 216 (78.3%) |  |  |  |
| Opportunity of improvement: n (%) | | |  |  | 1.4 | .498 |
| N-Miss | 3 | 1 | 4 |  |  |  |
| Not at all | 9 (5.5%) | 13 (8.3%) | 22 (6.8%) |  |  |  |
| A little | 22 (13.4%) | 24 (15.4%) | 46 (14.4%) |  |  |  |
| A lot | 133 (81.1%) | 119 (76.3%) | 252 (78.8%) |  |  |  |
| Decrease the attention towards the chronic disease: n (%) | | |  |  | 1.5 | .464 |
| Not at all | 96 (57.5%) | 88 (56.1%) | 184 (56.8%) |  |  |  |
| A little | 35 (21.0%) | 27 (17.2%) | 62 (19.1%) |  |  |  |
| A lot | 36 (21.5%) | 42 (26.7%) | 78 (24.1%) |  |  |  |
| To feel neglected: n (%) | | |  |  | 1.7 | .428 |
| N-Miss | 2 | 1 | 3 |  |  |  |
| Not at all | 147 (89.1%) | 143 (91.7%) | 290 (90.3%) |  |  |  |
| A little | 13 (7.9%) | 7 (4.5%) | 20 (6.2%) |  |  |  |
| A lot | 5 (3.0%) | 6 (3.8%) | 11 (3.4%) |  |  |  |
| To feel assisted as usual: n (%) | | |  |  | 2.6 | .273 |
| N-Miss | 4 | 4 | 8 |  |  |  |
| Not at all | 3 (1.8%) | 3 (2.0%) | 6 (1.9%) |  |  |  |
| A little | 12 (7.4%) | 5 (3.2%) | 17 (5.4%) |  |  |  |
| A lot | 148 (90.8%) | 145 (94.8%) | 293 (92.7%) |  |  |  |
| Note: Kruskall-Wallis H Test; Chi-Square χ^2^ Test. * p < .05; ** p < .001 | | | | | | |

| TABLE S5. Resilience Scale | | | |  |  |
| --- | --- | --- | --- | --- | --- |
| Characteristics | **Observed** | | **Total (N=324)** | **Kruskall-Wallis H Test** | **p-value** |
|  | PLWH (N=167) | PLWOD (N=157) |  |  |  |
| N-Miss | 1 | 2 | 3 |  |  |
| Resilience: mean (sd) | 59.7 (6.89) | 59.6 (7.0) | 59.7 (6.92) | - | .989 |
| Median (Q1, Q3) | 61.0 (55.2, 64.0) | 61.0 (56.0, 64.0) | 61.0 (56.0, 64.0) |  |  |
| Minimum | 24.0 | 25.0 |  |  |  |
| Maximum | 70.0 | 70.0 |  |  |  |
| Items of the RS |  |  |  |  |  |
| RS1 |  |  |  | 0.6 | 0.451 |
| N-Miss | 1 | 2 | 3 |  |  |
| Mean (sd) | 5.9 (1.1) | 6.0 (1.0) | 6.0 (1.1) |  |  |
| RS2 |  |  |  | 2.1 | 0.147 |
| N-Miss | 1 | 2 | 3 |  |  |
| Mean (sd) | 6.3 (1.1) | 6.5 (0.9) | 6.4 (1.0) |  |  |
| RS3 |  |  |  | 0.4 | 0.523 |
| N-Miss | 1 | 2 | 3 |  |  |
| Mean (sd) | 5.5 (1.4) | 5.6 (1.5) | 5.5 (1.4) |  |  |
| RS4 |  |  |  | 0.3 | 0.574 |
| N-Miss | 1 | 2 | 3 |  |  |
| Mean (sd) | 5.6 (1.6) | 5.8 (1.5) | 5.7 (1.5) |  |  |
| RS5 |  |  |  | 0.05 | 0.820 |
| N-Miss | 1 | 2 | 3 |  |  |
| Mean (sd) | 6.0 (1.2) | 6.0 (1.2) | 6.0 (1.2) |  |  |
| RS6 |  |  |  | 1.0 | 0.324 |
| N-Miss | 1 | 2 | 3 |  |  |
| Mean (sd) | 6.1 (1.0) | 6.2 (1.0) | 6.2 (1.0) |  |  |
| RS7 |  |  |  | 0.7 | 0.404 |
| N-Miss | 1 | 2 | 3 |  |  |
| Mean (sd) | 6.2 (1.0) | 6.1 (1.1) | 6.1 (1.0) |  |  |
| RS8 |  |  |  | 0.3 | 0.599 |
| N-Miss | 1 | 2 | 3 |  |  |
| Mean (sd) | 6.2 (1.3) | 6.3 (1.2) | 6.2 (1.3) |  |  |
| RS9 |  |  |  | 0.5 | 0.473 |
| N-Miss | 1 | 2 | 3 |  |  |
| Mean (sd) | 6.0 (1.0) | 6.0 (1.0) | 6.0 (1.0) |  |  |
| RS10 |  |  |  | 9.7 | 0.002* |
| N-Miss | 1 | 2 | 3 |  |  |
| Mean (sd) | 5.8 (1.4) | 5.3 (1.6) | 5.5 (1.5) |  |  |
| Note: Kruskall-Wallis H Test. * p < .05; ** p < .001 | | | | | |

**Fig. S1** Heatmap of the loadings estimated by the factor analysis. Values below 0.31 were set to 0. The factors represented on the x-axis are: F1=anxiety/concern; F2=psychological problems; F3=concern of the infection; F4=need of attention; F5=feelings of anger; F6=coping; F7=lockdown conditions. The variables displayed on the y-axis are: V1=number of rooms in the house; V2=psychological problems; V3=feeling calm; V4=feeling indifferent; V5=feeling sad; V6=feeling helpless; V7=feeling angry; V8=feeling nervous; V9=feeling restless; V10=feeling worried; V11=feeling anxious; V12=feeling scared; V13=feeling panicked; V14=feeling isolated; V15=manage the situation serenely; V16=signs of being unwell; V17=worried for economic problems; V18=feeling lonely; V19=apply meditative techniques; V20=take pleasure from activities; V21=keeping good relationships; V22=talk with family; V23=talk with other patients; V24=sleep quality; V25=feeling pessimist; V26=feeling confident; V27=eating more; V28=smoking; V29=alcohol consumption; V30=taking more drugs; V31=thinking about suicide; V32=helped by faith; V33=need to talk with a psychologist; V34=others more scared; V35=personal growth opportunity; V36=weight change; V37=feeling more at risk; V38=risk to worsen the disease; V39=worried to get COVID; V40= emotionally supported by doctors; V41=worried about doctors getting COVID; V42=worried about decreased attention by doctors; V43= feeling neglected; V44=feeling assisted


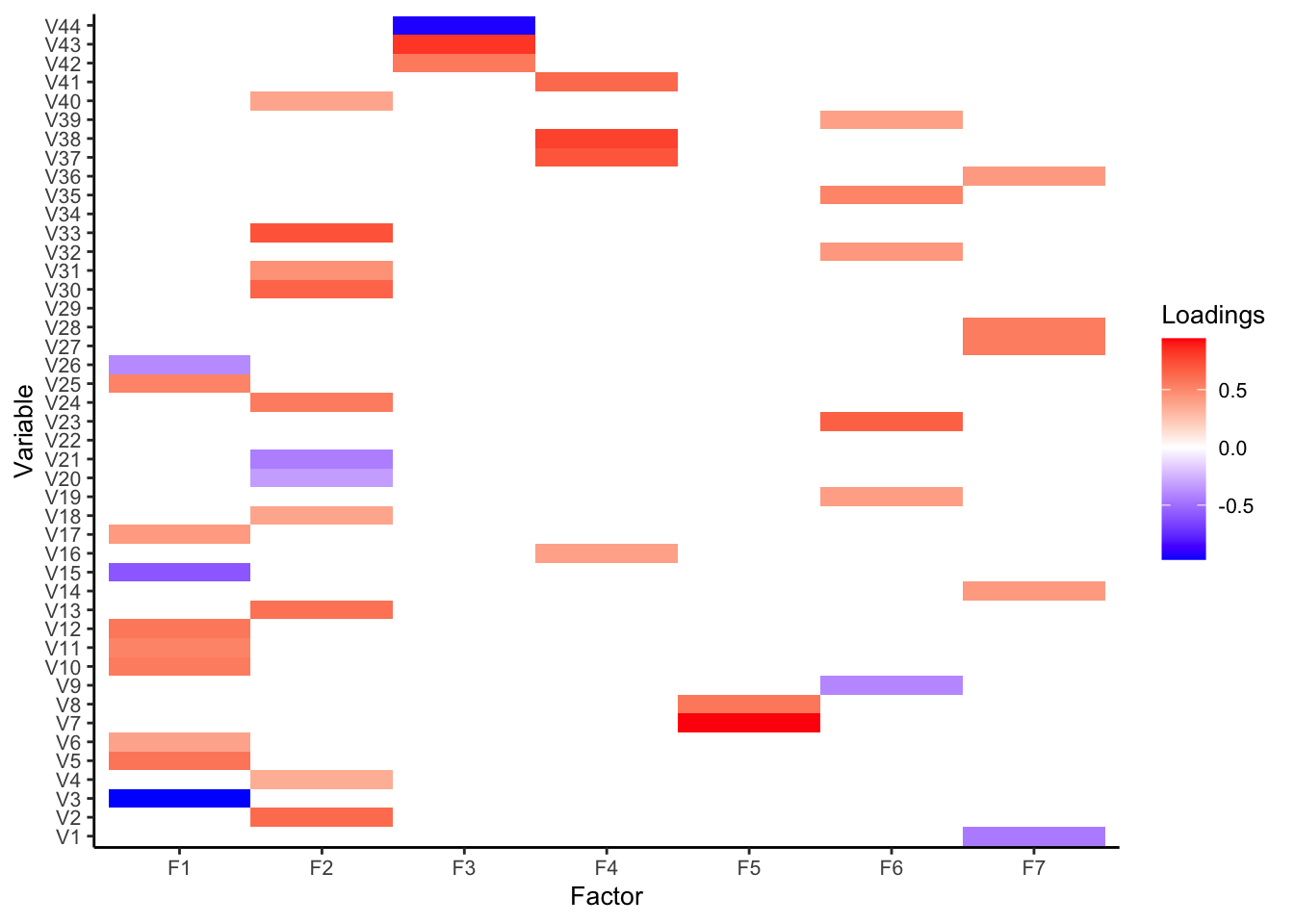


Fig.S1 Heatmap of the loadings estimated by the factor analysis

| TABLE S6. Linear regression analysis | | | |
| --- | --- | --- | --- |
| Resilience Score | | | |
| Predictors | Estimates | 95% CI | p-values |
| PLWOD vs PLWH | -1.00 | -3.37 – 1.38 | .410 |
| Male vs Female | 0.15 | -1.61 – 1.90 | .870 |
| Age | -0.03 | -0.12 – 0.06 | .508 |
| Foreigner vs Italian | 1.35 | -2.56 – 5.26 | .497 |
| Middle school vs primary school diploma | -1.04 | -3.6 – 1.51 | .421 |
| High school vs primary school diploma | -1.57 | -4.30 – 1.15 | .257 |
| Degree vs primary school diploma | -0.36 | -3.70 – 2.98 | .833 |
| Unemployed vs employed | -3.52 | -6.68 – -0.35 | .029***** |
| Housewife vs employed | -0.39 | -3.45 – 2.67 | .801 |
| Pensioners vs employed | 0.13 | -2.19 – 2.45 | .913 |
| Other occupation vs employed | 0.75 | -3.92 – 5.41 | .753 |
| With companionship vs single | 1.26 | -0.91 – 3.43 | .255 |
| Diagnosis > 5 years vs < 5 years | -0.33 | -2.42 – 1.76 | .759 |
| Feelings of anger | -1.76 | -2.67 – -0.85 | < .001****** |
| Need of attention | 0.00 | -0.88 – 0.89 | .996 |
| Concern of the infection | -0.32 | -0.83 – 0.19 | .213 |
| Psychological problems | -2.18 | -3.17 – -1.18 | < .001****** |
| Anxiety/concern | -1.56 | -2.30 – -0.82 | < .001****** |
| Coping | 0.48 | -0.47 – 1.44 | .322 |
| Lockdown conditions | 0.22 | -0.86 – 1.30 | .686 |
| Note: * p < .05; ** p < .001 | | | |
